# Adopting a logical framework model to help achieve a balanced and healthy vaccine R&D portfolio

**DOI:** 10.12688/wellcomeopenres.15168.2

**Published:** 2019-07-01

**Authors:** Bhoomi Lalani, Sourabh Sobti

**Affiliations:** 1 MSD Wellcome Trust Hilleman Labs Pvt. Ltd., New Delhi, New Delhi, 110062, India

**Keywords:** Vaccine, R&D, framework, portfolio, health, portfolio health, logical, Hilleman, evaluation framework

## Abstract

Vaccines are currently the 5th biggest therapy area with global sales for prophylactic and therapeutic vaccines to be ~ $30B, which is expected to increase to $45B by 2024. Immunization is globally recognized as one of the best investments to improve health, with impact lasting beyond saving 2-3M lives every year with benefits accrued over a lifetime.

Enterprise value of any R&D company is a cumulative sum of its projects and proprietary technologies. Hence organizations need to continuously evaluate their portfolios to review the health of projects as changes in external environment may impact project viability. Simultaneously, addition of any new project in a company’s portfolio is a significant investment and needs to be evaluated using an objective multi-parametric framework. In this pursuit, Hilleman Labs, an equal joint venture by MSD and Wellcome Trust, has created a logical framework to evaluate potential vaccine candidates before they are added to the portfolio.

## Introduction

Vaccines are currently the 5 ^th^ biggest therapy area by sales volume, with global sales for prophylactic and therapeutic vaccines ~ $30B ^1^, which is expected to increase to $45B ^1^ by 2024. Immunization is globally recognized as one of the best investments to improve health, with impact lasting much beyond saving 2-3M lives every year ^2^ with benefits accrued over a lifetime ^3^. This increase is due to inclusion of new vaccines in the Expanded Program of Immunization, especially in low- and middle-income countries supported by the Gavi, the Vaccine Alliance (Gavi) and global stakeholders, such as Bill & Melinda Gates Foundation, World Health Organization and United Nations International Children's Emergency Fund (UNICEF). In addition, increasing acceptance of the role of vaccines to fight pandemics and other infections, and reducing anti-microbial resistance (AMR), is also driving the growth of vaccine usage worldwide.

Given the importance of vaccines in improving public health, there are over 70 private, non-profit and public sector companies currently engaged in human vaccine development, with all firms having an appreciation that any new project/product they embark upon will require a gestation period of 10–12 years from laboratory to licensure ^4, 5^. Research involving novel products is iterative, requires significant investment and needs to account for attrition. A recent study pegs the cost of taking a biopharmaceutical from pre-clinical stage to licensure at $125M ^6^, with another estimating the risk adjusted cost of R&D between USD $130-350M ^5^. A more inclusive study by Tufts University estimates the out-of-pocket costs for development of a New Molecular Entity as ~ $1.4B ^4^.

Enterprise value of any R&D company is a cumulative sum of its projects and proprietary technologies. Hence organizations need to continuously evaluate their portfolios to review the health of projects as changes in external environment may impact project viability. Simultaneously, addition of any new project in a company’s portfolio is a significant investment and needs to be evaluated using an objective multi-parametric framework. In this pursuit, Hilleman Labs, an equal joint venture by MSD and Wellcome Trust, has created a logical framework to evaluate potential vaccine candidates before they are added to the portfolio.

The goal of this framework is to guide decision-making by focusing on multiple factors that would assist the organization in predicting capability and vision-fit resulting in a healthy portfolio.

### About Hilleman Labs

Hilleman Laboratories was established as an equal joint venture by MSD and Wellcome Trust in 2009, headquartered in New Delhi, India. Hilleman Laboratories is a global vaccine R&D organization committed to developing high impact, affordable vaccines for low- and middle- income countries. Our translational research focuses on creating safe, low-cost vaccines and innovative delivery technologies that are highly effective and can be easily incorporated into immunization programs. Hilleman Lab’s focus is on transforming ideas into products and technologies through translational R&D and by building partnerships with global stakeholders and vaccine manufacturers.

## Approach to creating the framework

Like most products and services, vaccine R&D candidates should be evaluated based on a series of internal and external factors that help an organization assess whether the molecule is in-line with the organization’s strengths, along with being relevant to the marketplace in the future.

A literature search was conducted to review and understand bio-pharma industry best practices to adding a new molecule in pipeline. We used key words such as “Vaccine research portfolio”, “vaccine R&D”, "vaccine portfolio”, and “portfolio management” etc. on databases available at Wellcome Open Research, Google Scholar and Google. We also leveraged the vaccine investment strategy framework used by Gavi ^a^ for selecting new vaccines to include in its portfolio, and perused strategic plans for public health institutes, such as Johns Hopkins Bloomberg School of Public Health, to gather information pertaining to industry practices.

The strategy team leveraged internal learning in terms of portfolio health evaluation and optimization to identify the factors important for Hilleman Lab’s performance and success. A cross-functional team, consisting of internal team leads for R&D, business development, manufacturing, quality and compliance, was constituted and convened to seek feedback on the framework to ensure the framework is comprehensive, objective and inclusive. The feedback cycle was a 2-step process. We developed a first draft of the framework, which was introduced to the team leaders in a meeting. There was a detailed discussion regarding inclusion/exclusion of parameters outlined in the draft framework. The weightage assigned to each individual factor was discussed to incorporate diverse points of view and make the framework adaptable by a wider audience within the organization.

The logical framework was then further refined and validated by utilizing data shared for potential vaccine candidates by research and development teams for a myriad of factors listed in Table 1. Once the changes were made to the framework, another meeting was convened to present the final version and gain buy-in.

**Table 1.** Logical Framework – Details.

Step 1			
Internal Factors			
	3 - High	2 - Medium	1 - Low
**Key capabilities / research** **expertise / critical knowledge**	▪ HL possesses technical expertise, critical knowledge and key capabilities (including trained staff, field of research) or has an identified partner with these capabilities	▪ HL possesses two of the three: technical expertise, critical knowledge and key capabilities (including trained staff, field of research) or has identified potential partner with these capabilities	▪ HL does not possess technical expertise, critical knowledge and key capabilities (including trained staff, field of research) and hasn't been able to identify a partner with these capabilities
**Portfolio fit**	▪The candidate/technology is perfect fit with current research projects (enteric diarrheal diseases and conjugate vaccine platform)	▪ The candidate/technology is not in complete alignment with current HL portfolio, but is still a fit within HL research activities	▪ The candidate/technology is completely different from past HL projects
**Resource fit**	▪ HL personnel are well-trained, experienced on the subject/topic of research	▪ HL personnel have some experience/know-how but would require additional training/support	▪ HL personnel have no prior knowledge of the field of research
**Personnel availability**	▪ Well-trained personnel with technical expertise are available and have spare capacity	▪ Well-trained personnel with technical expertise are available but have competing priorities (simultaneously working on other projects)	▪ Well-trained personnel with technical expertise are not available due to ongoing projects
**Ability to utilize current** **infrastructure**	▪ Research does not require any change/addition to existing infrastructure (equipment, work space, etc.) / there is an identified partner with the required infrastructure	▪ Research requires slight change/addition to existing infrastructure (equipment, work space, etc.) / there is potential to partner with someone with the required infrastructure	▪ Research requires complete overhaul of infrastructure / there is no potential partner with the required infrastructure
**Leveraging partnerships**	▪ HL will be able to leverage existing partnerships or has identified a partner, partner is/will be keen to collaborate	▪ HL may be able to leverage existing partnerships or is in the process of identifying a partner, unsure whether partner will be keen to collaborate	▪ HL does not have existing partnership/ collaborations in the field and is unable to identify a partner
**Public Health Impact**	▪ There is a pressing public health need in high, middle- and low-income countries, HL should pursue irrespective of other factors	▪ There is a moderate-high public health need in at least two of high/middle/low income countries HL should pursue irrespective of other factors	▪ There is a low public health need in any one of high/middle/low income countries
Step 2			
External Factors			
	3 - High	2 - Medium	1 - Low
**Mortality**	▪ High disease burden in terms of overall mortality, mortality in children <5 years	▪ Moderate disease burden in terms of overall mortality, mortality in children <5 years	▪ Low disease burden in terms of overall mortality, mortality in children <5 years
**Morbidity**	▪ High disease burden in terms of overall morbidity, QALY, etc.	▪ Moderate disease burden in terms of overall morbidity, QALY, etc.	▪ Low disease burden in terms of overall morbidity, QALY, etc.
**Supply vs Demand**	▪ Strong demand and less supply in the market, indicated by expected volume demand and anticipated revenue	▪ Moderate demand and supply in the market, indicated by expected volume demand and anticipated revenue	▪ Low demand and high supply in the market, indicated by expected volume demand and anticipated revenue
**No. of competitors**	▪ No competitors in the market currently	▪ 1-2 competitors in the market currently	▪ Many competitors in the market
**Vaccine Pipeline**	▪ Few other molecules in pipeline, in initial stages of development, low IP restrictions	▪ More than 4 molecules in pipeline, initial to advanced stages of development, moderate IP restrictions	▪ Many molecules in pipeline, some in advanced stages, high IP restrictions
**Market Pricing**	▪ High expected market pricing (hence, high revenue)	▪ Moderate expected market pricing (hence, moderate revenue)	▪ Low expected market pricing (hence, low revenue)
**Technical Feasibility**	▪ The project feasibility is high, considering technical, regulatory, IP, vaccine design etc.	▪ The project feasibility is medium considering technical, regulatory, IP, vaccine design etc.	▪The project feasibility is low, considering technical, regulatory, IP, vaccine design etc.
Step 3			
	3 - High	2 - Medium	1 - Low
**Antimicrobial Resistance**	▪ High risk of AMR	▪ Medium risk of AMR	▪ Low risk of AMR
**Improving Health Equity**	▪ High impact on improving health equity	▪ Medium impact on improving health equity	▪ Low impact on improving health equity
**Epidemic Potential**	▪ High epidemic potential	▪ Medium epidemic potential	▪ Low epidemic potential

**Figure 1.**
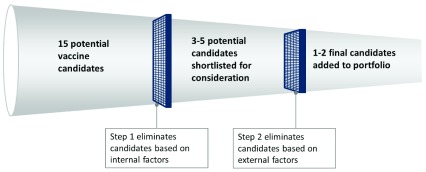
Evaluation framework. The figure captures the underlying principles and working of the evaluation framework by identifying two primary filters applied as a part of the process.

The logical framework can be further customized by organizations by incorporating/removing certain factors based on relevance. It is a two-step process that helps Hilleman Labs select a candidate(s) by eliminating potential candidates using a set of internal and external factors ( Figure 1; Table 2). The process of finalizing internal and external factors was informed by various aspects of the business. For internal factors, we considered key strengths of a vaccine R&D organization, including Hilleman Labs. In addition, we also looked at factors critical to achieving efficiencies, i.e. reducing costs by limiting new investments, ability to utilize current infrastructure and leveraging partnerships and ensuring public health impact in line with Hilleman Labs mission and vision. For external factors, primary determinants of supply and demand were included. In addition, to address economic considerations, we included factors, such as number of competitors, vaccine pipeline and market pricing and technical feasibility.

**Table 2.** Internal and external factors included in the framework.

Internal Factors (Step 1)	External Factors (Step 2)
Key capabilities / Research expertise / Critical knowledge	Mortality
Portfolio Fit	Morbidity
Resource Fit	Supply vs Demand
Resource Availability	Number of Competitors
Ability to Utilize Current Infrastructure	Vaccine Pipeline
Leveraging Partnerships	Market Pricing
Public Health Impact	Technical Feasibility

## The framework and how it works

Table 1 contains the detailed framework proposed.

The process of finalizing internal and external factors was informed by various aspects of the business.

For internal factors, we considered key strengths of a vaccine R&D organization, including Hilleman Labs.

*Organizational strengths:* To develop a successful vaccine candidate, R&D organizations should first explore internal factors that are its established key strengths. We have classified the internal factors under the following 4 categories:

- Knowledge: Technical aspects such as key capabilities (e.g. vaccine platforms), research expertise and critical knowledge play an important role in developing a strong vaccine portfolio. Being able to rely on internal expertise by emulating past successes and further strengthening the portfolio can be an indicator of the potential of a vaccine candidate.
- Resources: The most important resource at an organization’s disposal is its people. Talented employees with technical know-how and the right skillset give a boost to the probability of success of any R&D project. Facilities and infrastructure are the tools that an organization equips its human resources with, to deliver value. If the new vaccine candidate under consideration does not require any incremental changes to the existing infrastructure, it leads to cost-saving by avoiding new investments, as well as time-saving by being able to initiate and undertake the project immediately.
- Partnerships: Vaccine research and development is a time and resource intensive process. By developing and leveraging collaborative partnerships, organizations can enhance the probability of success. An organization engaged in vaccine development would require productive partnerships with multiple organizations across the development lifecycle.
- Vision/Mission: An organization’s vision and mission ensures that the activities associated with developing the new vaccine candidate are in-line with the organization’s goals and will contribute in achieving those goals. For Hilleman Labs, being able to create public health impact by developing vaccines for low- and middle-income countries is a crucial part of what we do. As a result, “public health impact” features as an internal factor for reviewing potential vaccine candidates.

Once the potential vaccine candidates have been evaluated based on the internal environment, in the second step we shift the focus to external factors impacting the outcome and uptake of a prospective vaccine. We have classified external factors under the following 3 categories:

- Epidemiology: Estimation of disease burden (morbidity, mortality etc.) is an important predictor of need for an intervention.
- Market Landscape: The interplay of supply vs demand, number of players in the market, number of candidates in the pipeline and expected unit price of the vaccine candidate play a crucial role in deciding economic success of a vaccine candidate. These are business considerations to guide management’s decision on adding a new candidate to the pipeline with a view to future sustainability.
- Technical Feasibility: This factor serves as the litmus test, as it is easy for teams to over-commit and embark on an overtly ambitious project. Hence this factor will reiterate that the potential new candidate is technically achievable.

For each of the internal and external factors, the evaluator is required to score the vaccine candidate as High (score=3), Medium (score=2) or Low (score =1). A clear definition of high, medium and low-ranking candidate is provided for all the factors to avoid subjective interpretation; with weights assigned to each internal and external factor to reflect relative importance. A candidate is evaluated against internal factors and a weighted average is calculated. Candidates with a weighted average above a threshold (0.5) progress to the next stage and are then assessed based on external factors. For details regarding the high, medium and low-ranking scores for all factors, please refer to Table 1.

At the end of Step 2, additional filters such as reducing AMR, improving health equity, epidemic potential, etc. are applied with only top 1 or 2 candidates being finally added to the portfolio ^3, 7, 8^. Vaccines help reduce AMR burden by protection against direct transmission of drug-resistant infections and simultaneously reducing the probability of illness and limit the prescription of antibiotics which is driving drug resistance in various pathogens ^9^. Vaccines also play a critical role in improving social and economic development and reducing health inequity and hence antigens targeting marginalized populations are prioritized ^10^. Focusing efforts towards developing a vaccine for diseases with a high epidemic potential, with no approved vaccine or insufficient accessibility to high risk population is another important criterion ^11^. These additional filters are based on emerging public health trends, which can be addressed by availability of quality assured vaccines. These factors may evolve with time as new threats emerge, resulting in a change in global priorities.

The framework provides an objective definition of high, medium and low categories, which helps the evaluator refrain from using prior knowledge and biases while ranking the candidates. For example, the framework defines “high” public health impact as “There is a pressing public health need in high, middle- and low-income countries, organization should pursue irrespective of other factors”. Since the definition clearly emphasizes a pressing need in high-, middle- and low-income countries, the evaluator is not tempted to score high or low based on pressing need in a specific patient population versus wider global impact. A distinct definition of each of the internal and external factors is provided in the framework, which reduces any biases or prejudices.

## Validation of the framework

**Figure 2.**
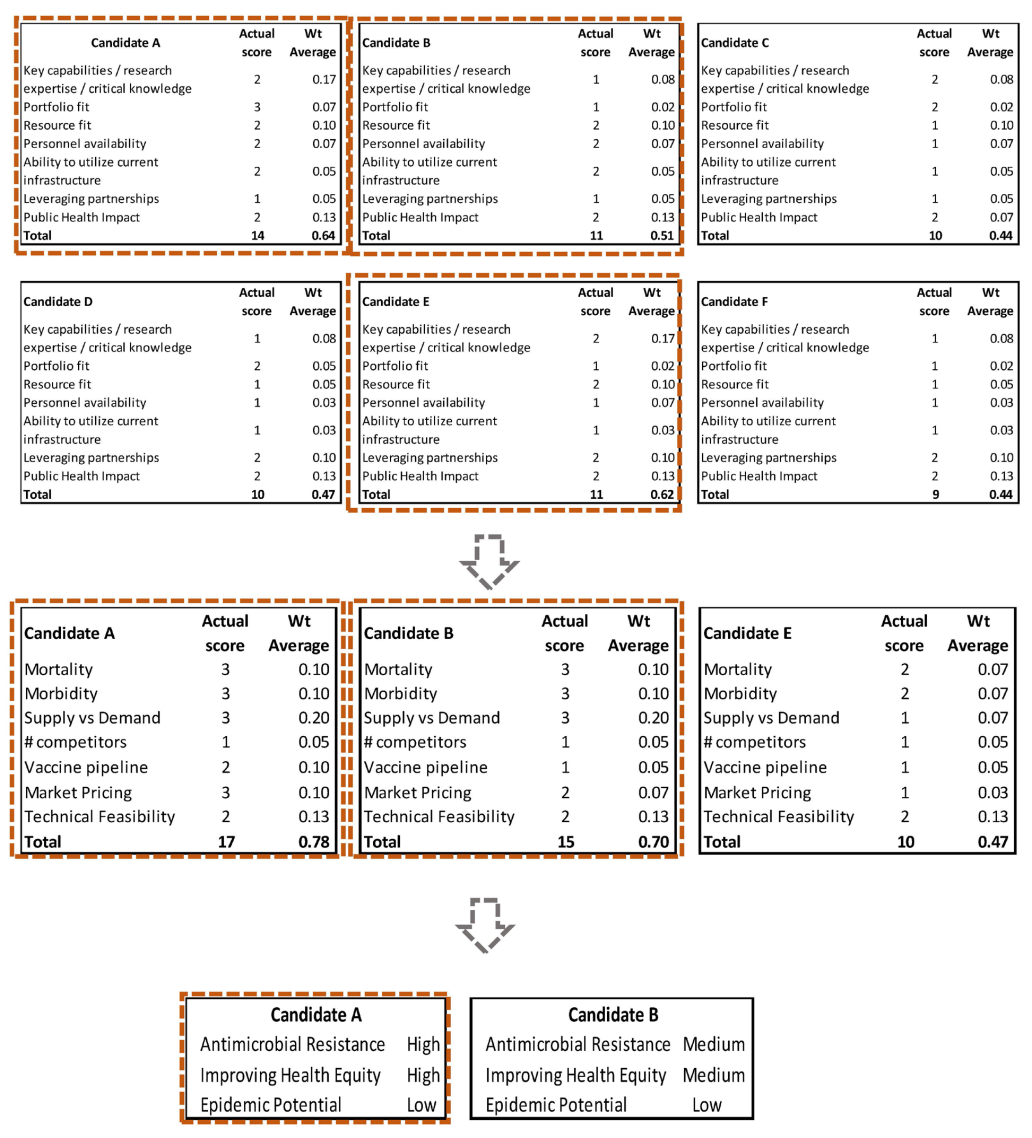
Logical Framework – Execution.

To validate and refine the framework, we evaluated 6 potential vaccine candidates (A-F) using the framework. The first step eliminated 3 candidates based on internal factors and the remaining passed to stage 2 of evaluation based on external factors. Post the stage 2 review, one candidate emerged as the top choice, which was then included in company’s development portfolio. For details see Figure 2.

## Conclusion

The pursuit of new products feels haphazard and sporadic for a vaccine research organization since the time from research to market is > 10 years ^4, 5^. Thus it becomes critical for organizations to evaluate a myriad of factors before adding a project to their portfolio. A more balanced and healthier portfolio will have products that feature across the spectrum of the development lifecycle (i.e. a mix of early and late stage candidates). General learning from this paper and entire exercise can be listed as follows:

- The primary consideration of an organization evaluating potential R&D candidates is internal strengths and weaknesses. This ensures rationality in decision-making as organizations are not tempted to embark upon an unfeasible journey, simply influenced by external/commercial attractiveness.
- Once internal capabilities are confirmed and aligned, the next step is to consider external pull. Different organizations are motivated by different external factors (e.g. commercial opportunity, unmet health need, etc.) and organizations could modify the framework by addition/deletion of factors or tweaking weightages assigned to each factor to customize the framework to suit their respective organization needs.
- Ultimately, for vaccine R&D organizations, rising public health burden owing to AMR, health inequities and epidemic potential are drivers of unmet need and should be taken into consideration as a final check before deciding to pursue a candidate.

The framework can then be used to objectively assess and evaluate multiple candidates before adding a candidate in an organization’s pipeline. Organization can also use the framework to review existing portfolio and re-prioritize candidates accordingly. As companies strive to develop a robust pipeline of vaccines across many infectious disease targets, a common pitfall is that they may be spreading their resources too thin; with increasing complexity and rising costs of conducting clinical trials over the years. Also, as a large number of companies are chasing similar vaccine targets resulting in affordable and competitive pricing, it simultaneously poses pressure on companies to strive for lower cost of goods and overall company sustainability. Hence, it is critical for vaccine development companies to review and manage their portfolio by allocating resources differentially, while simultaneously terminating nonviable projects to ensure product relevance in the ever-evolving disease and global market landscape.

## Data availability

No data is associated with this article.
