# Supplementary material for: Adopting a logical framework model to help achieve a balanced and healthy vaccine R&D portfolio
Source: Wellcome Open Res. 2019 Jul 1;4:64. Originally published 2019 Apr 3. [Version 2] doi: 10.12688/wellcomeopenres.15168.2 (PMC6625608; doi:10.12688/wellcomeopenres.15168.2)
Supplement: Peer review history

# Peer review: Reviewer response for version 2

**DOI:** 10.21956/wellcomeopenres.16737.r35900

**Published:** 2019-07-11

**Authors:** Gavin Yamey

**Affiliations:** 1 Global Health Institute, Duke University, Durham, NC, USA; 2 Center for Policy Impact in Global Health, Duke University, Durham, NC, USA

The authors have responded to my suggestions, with the exception of one - they are unable to give examples of how they used this logical framework model in actual practice due to proprietary/confidentiality concerns. This is understandable but unfortunate for readers and for learning!

I confirm that I have read this submission and believe that I have an appropriate level of expertise to confirm that it is of an acceptable scientific standard.

# Peer review: Reviewer response for version 1

**DOI:** 10.21956/wellcomeopenres.16549.r35220

**Published:** 2019-06-03

**Authors:** Gavin Yamey

**Affiliations:** 1 Global Health Institute, Duke University, Durham, NC, USA; 2 Center for Policy Impact in Global Health, Duke University, Durham, NC, USA

This short open letter from Hilleman Laboratories (an MSD-Wellcome Trust joint venture) describes a framework that the company uses to help prioritize its investments. As shown in Figure 1 and Table 2, out of a list of potential vaccine candidates, the company uses various criteria to decide which ones to pursue.

 While it is certainly of interest for readers to learn about the internal decision-making processes of a vaccine development company, at the end of this letter, I was left wondering about a number of questions. I think that if the authors were able to address these questions, this open letter would be strengthened.

 First, what are the generalizable lessons from Hilleman Laboratories’ internal framework? In other words, for researchers, companies, PDPs, etc., what are the lessons from this framework?

 Second, and this question relates to the first question, what impact has using this framework had? How can readers judge its success? For example, did adoption of this framework lead to a higher rate of product launches?

 In addition to these questions, I have one other major comment and some minor comments.

 Major comment: it would be very helpful indeed if you could give actual examples of how this framework was applied. This would help “bring alive” the use of this framework.

 Minor comments:

- Abstract and opening sentence: does “5 ^th^ biggest therapy area” refer to sales volumes?
- Introduction: Gavi is now called “Gavi, the Vaccine Alliance”.
- Under public health institutes, it may be appropriate to say “Johns Hopkins Bloomberg School of Public Health” rather than Johns Hopkins Medicine.
- Why did you only search Wellcome Open Research and Google? This narrow search strategy could have missed several relevant documents.
- In terms of the criteria used for decision-making, I was surprised that there is no consideration of whether the disease has “missing” tools. There are several high-burden conditions (e.g. TB) where new tools (in this case a highly effective preventive vaccine) are desperately needed.

I confirm that I have read this submission and believe that I have an appropriate level of expertise to confirm that it is of an acceptable scientific standard, however I have significant reservations, as outlined above.

# Response

**Published:** 2019-06-07

**Authors:** Bhoomi Lalani

**Affiliations:** MSD Wellcome Trust Hilleman Labs, India

Thank you for your detailed and insightful comments. Please see below for responses to your suggestions to strengthen the article.

1. First, what are the generalizable lessons from Hilleman Laboratories’ internal framework? In other words, for researchers, companies, PDPs, etc., what are the lessons from this framework?

**Response**: General learning from this paper and entire exercise can be listed as follows:

The primary consideration of an organization evaluating potential R&D candidates is internal strengths and weaknesses. This ensures rationality in decision-making as organizations are not tempted to embark upon an unfeasible journey, simply influenced by external/commercial attractiveness.

- Once internal capabilities are confirmed and aligned, the next step is to consider external pull. Different organizations are motivated by different external factors (e.g. commercial opportunity, unmet health need, etc.) and organizations could modify the framework by addition/deletion of factors or tweaking weightages assigned to each factor to customize the framework to suit their respective organization needs.
- Ultimately, for vaccine R&D organizations, rising public health burden owing to AMR, health inequities and epidemic potential are drivers of unmet need and should be taken into consideration as a final check before deciding to pursue a candidate.

We will include key learning in the conclusion section of the open letter.

2. Second, and this question relates to the first question, what impact has using this framework had? How can readers judge its success? For example, did adoption of this framework lead to a higher rate of product launches?

**Response**: In the long term, we do sincerely hope that adoption of this framework will improve efficiency and productivity as companies will allocate resources to discerning candidates; however as it takes ~ 10 years from pre-clinical to licensure this can only be assessed at a much later date.

3. Major comment: it would be very helpful indeed if you could give actual examples of how this framework was applied. This would help “bring alive” the use of this framework.

**Response**: The primary reason we did not disclose the scores as assessment on Internal factors reflects the  Hilleman core capabilities and we would like to maintain confidentiality around them.

Minor comments:

- Abstract and opening sentence: does “5 ^th^ biggest therapy area” refer to sales volumes? **Response**: Yes, it does.
- Introduction: Gavi is now called “Gavi, the Vaccine Alliance”. Response: Thank you, will update.
- Under public health institutes, it may be appropriate to say “Johns Hopkins Bloomberg School of Public Health” rather than Johns Hopkins Medicine. **Response**: Thank you, will update.
- Why did you only search Wellcome Open Research and Google? This narrow search strategy could have missed several relevant documents. **Response**: We primarily searched Wellcome Open Research, Google and Google Scholar and agree this may have precluded certain documents.
- In terms of the criteria used for decision-making, I was surprised that there is no consideration of whether the disease has “missing” tools. There are several high-burden conditions (e.g. TB) where new tools (in this case a highly effective preventive vaccine) are desperately needed. **Response**: Companies can tweak one of the factors to assign higher weightage for disease with “missing tools”

# Peer review: Reviewer response for version 1

**DOI:** 10.21956/wellcomeopenres.16549.r35219

**Published:** 2019-04-23

**Authors:** Jayasree K. Iyer

**Affiliations:** 1 Access to Medicine Foundation, Amsterdam, The Netherlands

The article is original and balanced in view. The authors have presented here a method, they have developed to assess the strength or "health" as they call it of a vaccine R&D portfolio. This is intended to be helpful for any organization, private or public in nature, to be able to evaluate the opportunity they have in addressing access while remaining a sustainable entity. In my view, this framework is indeed useful and will inform readers well. The paper is well aligned with today's needs and the international agenda on vaccines access.

 A few suggestions to strengthen the article are here:

- Demand drivers may need to be more detailed as the purchasing of any vaccine and how it is used would influence the strength of each parameter (the high, medium low-ness).
- A few example products and diseases especially in Figure 2 to help illustrate the candidates and why the "scores" are given as such would have greatly helped the reader understand the application of the framework, and increases its usability.
- It is useful to spell out how addressing resistance (AMR) helps the framework, as this is often not well understood. A few example of the fact that reducing usage of antibiotics, lowering infection rates, etc as not all vaccine candidates truly has the potential to reduce AMR, unless a threshold of effectiveness is achieved (that may require some caveats and detail as a next step). 
- The authors could also illustrate how single candidates addressing single infections versus combination vaccines (multiple serotypes of a single infectious agent or multiple diseases within a vaccine) will have a different strength to a vaccine portfolio (covering more diseases will definitely outweigh a single disease candidate). Illustrating that could be helpful if space allows.

Apart from that, a very interesting read and well done!

I confirm that I have read this submission and believe that I have an appropriate level of expertise to confirm that it is of an acceptable scientific standard.

# Response

**Published:** 2019-05-01

**Authors:** Bhoomi Lalani

**Affiliations:** MSD Wellcome Trust Hilleman Labs, India

Thank you for your detailed and insightful comments. Please see below for responses to your suggestions to strengthen the article.

1. Demand drivers may need to be more detailed as the purchasing of any vaccine and how it is used would influence the strength of each parameter (the high, medium low-ness).

**Response:** We do agree for a vaccine to be accessible to the end user, procurement and supply chain management is critical. Several global organizations such as UNICEF, PAHO, GAVI, etc. are actively involved in purchasing and distribution of vaccines to low- and middle-income countries. However, as a vaccine R&D organization, first consideration is the need for a vaccine. As time from lab to licensure for a novel vaccine will take ~ 8-10 years, demand dynamics may shift over the years and hence we have considered morbidity/mortality as a measure to assess need rather than actual procurement volumes.

2. A few example products and diseases especially in Figure 2 to help illustrate the candidates and why the "scores" are given as such would have greatly helped the reader understand the application of the framework and increases its usability.

**Response: **Agree **- **Figure 2 aims to illustrate why the scores are what they are, by referring to the definitions of high, medium, low in the table. However, since the scores featuring in Figure 2 are weighted average (based on relative importance) and readers are not provided information on weights assigned to individual factors and the calculation of weighted average, direct correlation of scores to definitions is not intuitive. We will revise the table 2 to reflect actual scores along with weighted average.

3. It is useful to spell out how addressing resistance (AMR) helps the framework, as this is often not well understood. A few examples of the fact that reducing usage of antibiotics, lowering infection rates, etc. as not all vaccine candidates truly has the potential to reduce AMR, unless a threshold of effectiveness is achieved (that may require some caveats and detail as a next step). 

**Response: **The article outlines the use of Anti-microbial resistance (AMR), along with additional 2 factors - improving health equity and epidemic potential at the 3rd step for assessing vaccines before including in company portfolio. We will revise the article to elaborate on these factors.

4. The authors could also illustrate how single candidates addressing single infections versus combination vaccines (multiple serotypes of a single infectious agent or multiple diseases within a vaccine) will have a different strength to a vaccine portfolio (covering more diseases will definitely outweigh a single disease candidate). Illustrating that could be helpful if space allows.

**Response: **Vaccine development is a lengthy process with probability of success ranging from 3%-9% from pre-clinical to licensure for novel candidate. Thus, the probability of success for a combination vaccine will be even lower, and while some companies are simultaneously pursuing development of 2 novel antigens in a combination vaccine; the chances of success will be less than 1%, hence will be a high risk proposition for any organization.
